# Accounting for Red Cell Distribution Width Improves Risk Stratification by Commonly Used Mortality/Deterioration Risk Scores in Adult Patients Hospitalized Due to COVID-19

**DOI:** 10.3390/life14101267

**Published:** 2024-10-05

**Authors:** Ana Jordan, Vladimir Trkulja, Ivana Jurin, Sanja Marević, Lovorka Đerek, Ivica Lukšić, Šime Manola, Marko Lucijanić

**Affiliations:** 1 Cardiology Department, University Hospital Dubrava, 10000 Zagreb, Croatia; 2 School of Medicine, University of Zagreb, 10000 Zagreb, Croatia; 3 Clinical Department for Laboratory Diagnostics, University Hospital Dubrava, 10000 Zagreb, Croatia; 4 School of Medicine, Catholic University of Croatia, 10000 Zagreb, Croatia; 5 Maxillofacial Surgery Department, University Hospital Dubrava, 10000 Zagreb, Croatia; 6 School of Dental Medicine, University of Zagreb, 10000 Zagreb, Croatia; 7 Hematology Department, University Hospital Dubrava, 10000 Zagreb, Croatia

**Keywords:** anisocytosis, inflammation, mortality, SARS-CoV-2, COVID-19

## Abstract

Higher red blood cell distribution width (RDW) levels have gained attention in the prognostication of many chronic metabolic and malignant diseases, as well as coronavirus disease 2019 (COVID-19). We aimed to evaluate whether accounting for RDW might contribute to risk stratification when added to commonly used risk scoring systems in adult COVID-19 patients. We retrospectively analyzed a cohort of 3212 non-critical COVID-19 patients hospitalized in a tertiary-level institution from March 2020 to June 2021. Admission RDW values were considered normal if they were ≤14.5% in males or ≤16.1% in females. The Modified Early Warning Score (MEWS), International Severe Acute Respiratory and Emerging Infections Consortium Coronavirus Clinical Characterisation Consortium score (ISARIC 4C), and Veterans Health Administration COVID-19 (VACO) index were evaluated as prognostic scores. RDW exceeded the upper limit in 628 (19.6%) of the patients. When RDW was accounted for, risks of the predicted outcomes were considerably different within the same MEWS, 4C score, and VACO index levels. The same patterns applied equally to patients who started, and those who did not start, remdesivir before deterioration. RDW may be a useful tool for stratifying risk when considered on top of commonly used prognostic scores in non-critical COVID-19 patients.

## 1. Introduction

Coronavirus disease 2019 (COVID-19), caused by severe acute respiratory syndrome coronavirus 2 (SARS-CoV-2), is an acute systemic inflammatory disease predominantly presenting with respiratory symptoms [1,2]. One of the main challenges imposed by the COVID-19 pandemic is the disruption of health services, with increasing demand for testing capacity, hospital beds, and medical equipment [3]. The endemic circulation of SARS-CoV-2 is likely to continue imposing a significant disease burden in the future [4]. To reduce the burden on the healthcare system and provide adequate care to patients simultaneously, predicting the clinical course of infection is of particular importance. Many prognostication models that were shown to be useful during the COVID-19 pandemic were re-utilized from other medical contexts. However, in addition to established prognostic models, additional biomarkers are often needed for the proper stratification of patients suffering from COVID-19.

Red blood cell distribution width (RDW), calculated by dividing the standard deviation of corpuscular volume by the mean corpuscular volume, is a parameter of the hemogram used in the differential diagnosis of anemia and involves variability in the form and size of red blood cells in the subject [5]. Previous studies have confirmed an association between high RDW and mortality in patients with coronary disease, liver disease, pancreatitis, ischemic stroke, and sepsis [6,7,8,9,10,11]. Although some studies have aimed to determine the association of elevated RDW with adverse prognosis in COVID-19, its usefulness has not been well established [12,13].

Therefore, in our clinical research, we aimed to evaluate whether accounting for RDW might contribute to risk stratification by commonly used risk scoring systems in adult COVID-19 patients: the Modified Early Warning Score (MEWS), which predicts the risk of death or intensive care unit (ICU) transfer within 60 days [14]; the Coronavirus Clinical Characterisation Consortium core (4C), developed by the International Severe Acute Respiratory and Emerging Infections Consortium (ISARIC), which predicts the risk of in-hospital mortality [15]; and the Veterans Health Administration COVID-19 (VACO) index, which predicts 30-day mortality risk [16].

## 2. Materials and Methods

### 2.1. Study Outline

Between 25 March 2020 and 8 June 2021, our institution was the main center for the treatment of COVID-19 patients. This included a respiratory center (a “regular” ward and ICU) specifically for patients with COVID-19 as the primary condition, but also the management of patients positive for SARS-CoV-2 infection who required other medical or surgical care and had to be isolated from other patients (i.e., COVID-19 was not the primary health issue). All patients had a positive polymerase chain reaction or rapid antigen COVID-19 test prior to hospital admission. All patients were adults and of the white race. Patients were treated according to contemporary guidelines, with the majority receiving low-molecular-weight heparin (LMWH) thromboprophylaxis and corticosteroids and with varying exposure to other drugs like remdesivir [17]. The clinical and laboratory data used in this paper are part of a hospital registry project. Data were 100% complete regarding the investigated variables. RDW on admission was expressed as a coefficient of variation (%) of mean corpuscular volume (MCV), as reported by the Advia 2120i automated cell counter (Siemens Medical Solutions Diagnostics Pte Ltd., Swords, Ireland). COVID-19 severity at admission was graded according to the World Health Organization (WHO) recommendations and national guidelines as mild, moderate, severe, or critical [18]. Mortality and other clinical outcomes were assessed from the start of the hospital stay. The MEWS [14], 4C score [15], and VACO index [16] were used as prognostic risk scores.

**Figure 1:**
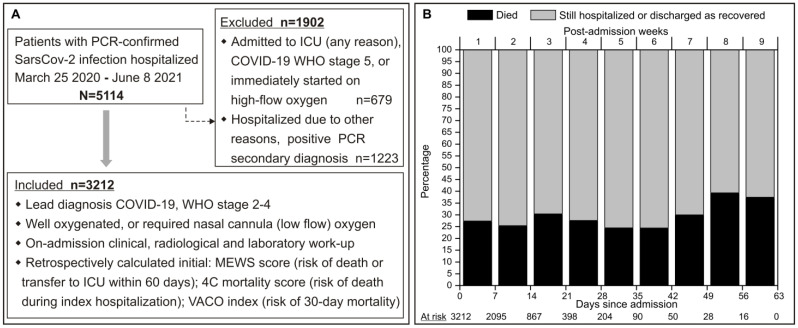
(**A**) Study outline. (**B**) Probability of dying by week of hospitalization (entire cohort). A complementary log–log model for continuous time process was fitted to probability of dying with time measured in weeks. 4C—Coronavirus Clinical Characterisation Consortium; ICU—intensive care unit; MEWS—Modified Early Warning Score; PCR—polymerase chain reaction; VACO—Veterans Health Administration COVID-19 index; WHO—World Health Organization.

Data generated through a detailed clinical, radiological, and laboratory work-up undertaken within approximately 12 h of hospital admission and stored in the institutional information system were used to retrospectively calculate the following: (i) on-admission WHO COVID-19 severity level; (ii) on-admission MEWS, 4C score, and VACO index. In line with the purpose of risk scoring systems, the present analysis is restricted to patients in whom COVID-19 was the primary diagnosis (reason for hospitalization) and the disease was mild to severe but not “critical” (Figure 1A). The frequencies of outcomes predicted by the three risk scoring systems (risk of death or ICU transfer within 60 days for MEWS, in-hospital mortality for the 4C score, and 30-day mortality for the VACO index), as well as the need for ICU transfer and mechanical ventilation (MV), are reported and evaluated with respect to RDW level: (i) within the normal range or (ii) above the normal range. Red cell distribution width was considered normal if it was ≤14.5% in male patients or ≤16.1% in female patients [19].

The analysis was conducted using anonymized data and was approved by the Institutional Ethics Committee.

### 2.2. Statistical Analysis

For each of the three scoring systems, the predicted outcomes (death or ICU transfer within 60 days for MEWS; in-hospital mortality for the 4C score; and 30-day mortality for the VACO index) and other outcomes are tabulated across their risk levels—overall and by RDW level (normal or >normal, i.e., “high”). The normality of the distribution of numerical variables was tested using the Kolmogorov–Smirnov test. Due to the non-normal distribution of all numerical variables, they are presented as medians and 25th–75th percentile ranges, and the non-parametric Mann–Whitney U test and Kruskal–Wallis ANOVA were used to compare them between subgroups. Categorical variables were presented as percentages and compared between subgroups using the chi-squared test. Predictive properties of the scores with and without accounting for RDW were assessed through area under the curve (AUC) values and respective 95% confidence intervals (CIs) provided by univariate and multivariate logistic regression analyses. To illustrate the dynamics of dying/cure–discharge over the weeks of hospitalization, a complementary log–log model for continuous time processes was fitted to the probability of dying, with time expressed in weeks. *p* values <0.05 were considered statistically significant. No formal power analyses were conducted due to the inclusion of a large number of consecutive patients from a single institution registry encompassing more than 3000 patients. We used SAS 9.4 for Windows software (SAS Inc., Cary, NC, USA) and MedCalc statistical software version 23.0.2 (MedCalc Software Ltd., Ostend, Belgium).

## 3. Results

### 3.1. Patient Eligibility and Characteristics

A total of 5114 adult patients were hospitalized during the observed period, 3212 (62.8%) of whom were included in the present analysis (Figure 1A). The longest hospitalization for a discharged cured patient, as well as the longest one for those who died during the index hospitalization, was 63 days (9 weeks). Weekly mortality for those at risk at the start of each subsequent week was consistently between 25% and 36% (Figure 1B). Patients were predominantly older (median 72 years) (Table 1), comparably men (56.2%) and women, almost exclusively (96.5%) with radiological evidence of pneumonia, mostly immediately started on low-flow oxygen (92.2%), and 17.6% were treated with remdesivir before disease progression (Table 1). RDW was beyond the upper limit in 628 (19.6%) of the patients (Table 1).

The proportion of patients with MEWSs 0–2 (7.9% predicted risk of death or ICU transfer within 60 days) was slightly higher (54.9%) than of patients with MEWSs 3–4 (12.7% predicted risk) (Table 1). In respect to the 4C score, most patients were scored as “high risk” (31.4–34.9% predicted mortality) (54.0%), followed by “medium risk” (9.1–9.9% predicted mortality) (25.3%) (Table 1). With respect to the VACO index 30-day mortality risk, 32.9% of patients were graded as “extreme risk” patients (≥21.3% predicted risk), while the prevalence of those with “low–medium–high” risks was 20.9–24.2% (Table 1). Overall, 12.6% were eventually transferred to the ICU, 10.8% were mechanically ventilated, 27.4% died during the index hospitalization, 26.2% died within 30 days, and 29.2% either died or required ICU treatment (Table 1).

As compared to those with RDW within the normal range, patients with RDW > normal (Table 1) were statistically significantly more likely to be older, were more commonly men and had diabetes mellitus, chronic heart failure, chronic kidney failure, and higher CCI, and were less frequently started on remdesivir before deterioration (*p* < 0.05 for all comparisons). They were also more likely to belong to lower-risk MEWS but higher-risk 4c score and VACO index categories (Table 1), and were more likely to be transferred to the ICU, require mechanical ventilation, and die (*p* < 0.05 for all comparisons).

### 3.2. Accounting for RDW Improves MEWS Scoring System-Based Risk Stratification

**Figure 2:**
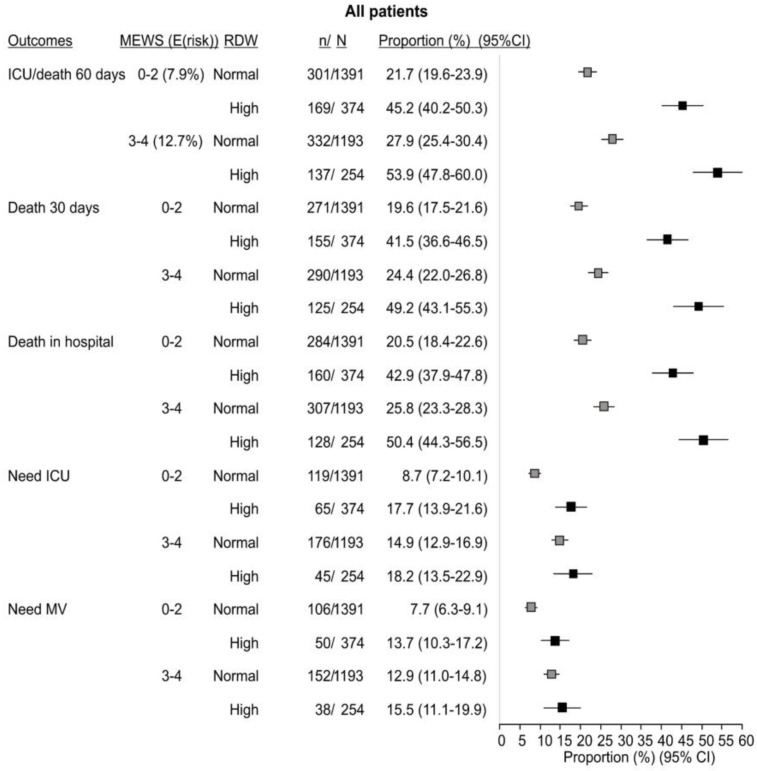
Probability of “death or intensive care unit (ICU) transfer within 60 days” predicted by the Modified Early Warning Score (MEWS) and probability of other outcomes by MEWS level (0–2 or 3–4) in respect to red cell distribution width (RDW). Probabilities (proportions) are given with Wilson 95% confidence intervals. MEWS risk levels are depicted by the MEWS and expected (E(risk)) probability associated with the respective score. MV—mechanical ventilation.

Patients with MEWSs 0–2 (7.9% predicted risk of death or ICU transfer within 60 days) and those with MEWSs 3–4 (12.7% predicted risk) significantly differed regarding a range of characteristics, including the proportion of those with RDW >normal and distribution across the risk levels based on the 4C and VACO scoring systems, and higher-risk patients were more likely to receive remdesivir (Supplementary Table S1, *p* < 0.05 for all comparisons). The risk of ICU transfer or death (outcome predicted by the MEWS) was only slightly but significantly higher in patients with scores 3–4 than in patients with scores 0–2—32.4% vs. 26.6%—and both were considerably higher than expected based on the MEWS (Supplementary Table S1). All other unfavorable outcomes were also slightly but significantly higher in MEWS 3–4 than in MEWS 0–2 patients (Supplementary Table S1). However, when RDW was accounted for, risks of the predicted outcome were considerably different within the same MEWS level (Figure 2): (i) if MEWS 0–2 and RDW normal, risk of death or ICU transfer was 21.7%, but it was 45.2% if RDW >normal; (ii) the same was true if MEWS 3–4 (27.9% if RDW normal vs. 53.9% if RDW >normal); (iii) the same pattern applied equally in patients started and those not started on remdesivir before deterioration (Supplementary Figure S1). In agreement, all mortality outcomes (death in 30 days, in-hospital mortality) followed the same pattern overall and in remdesivir-started and not-started patients (Figure 2 and Supplementary Figure S1). Proportions of those transferred to the ICU or requiring mechanical ventilation were also higher in patients with high vs. normal RDW at each level of MEWS/remdesivir treatment, but differences were small, i.e., not nearly as profound as in the case of the mortality outcomes (Figure 2). When investigating predictive properties of MEWS, it provided overall modest predictive properties of the investigated outcome (AUC 0.556, 95% CI (0.539–0.574)) when used as a sole parameter. However, its properties were significantly improved when additionally accounting for elevated RDW (AUC 0.632, 95% CI (0.615–0.649)).

### 3.3. Accounting for RDW Improves 4C Score-Based Risk Stratification

**Figure 3:**
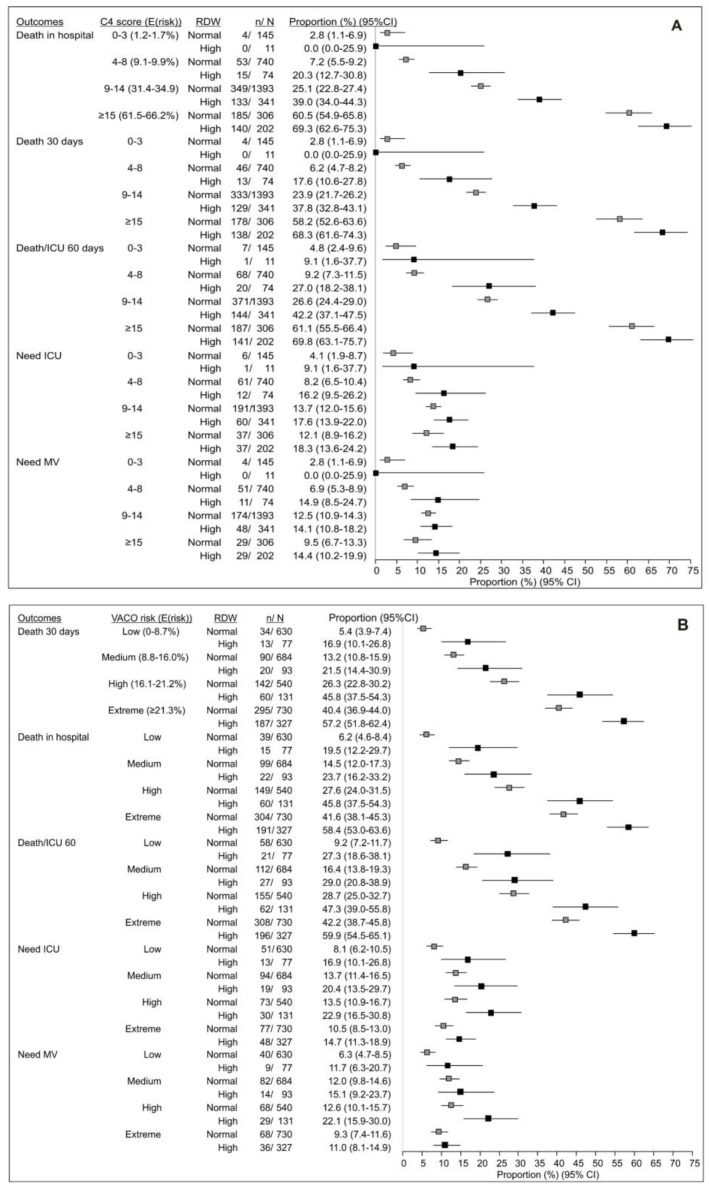
(**A**) Probability of in-hospital death predicted by the 4C score and probability of other outcomes across the 4C risk levels in respect to red cell distribution width (RDW). 4C risk levels are depicted by the score and expected (E(risk)) probability associated with the respective score. (**B**) Probability of 30-day mortality predicted by the VACO index (Veterans Health Administration COVID-19 index) and probability of other outcomes across the VACO risk levels in respect to RDW. VACO risk levels are depicted by the risk level and expected (E(risk)) probability associated with the respective level. Probabilities (proportions) are with Wilson 95% confidence intervals. ICU—intensive care unit; MV—mechanical ventilation.

Across the 4C levels of risk of in-hospital mortality (“low” [1.2–1.7%] to “very high” [61.5–66.2%]), patients were significantly more likely to be progressively older (Supplementary Table S2), more commonly with obvious pneumonia, less commonly started on remdesivir before further deterioration, and more commonly had high RDW and higher prevalence of higher-scored VACO 30-day mortality risks (*p* < 0.05 for all analyses). In-hospital mortality in the lowest C4 risk level (“low risk”, score 0–3) was 2.6%, which was close to the expected mortality (1.2–1.7%); it was 8.4% at the “medium risk” (score 4–8) level, in line with expectations (9.1–9.9%); it was 27.8% at the “high risk” (score 9–14) level, again in line with the expectation (31.4–34.9%); and it was 64.0% in the “very high risk” level (expected—61.5–66.2%) (Supplementary Table S2). All other outcomes (30-day mortality, ICU transfer, mechanical ventilation, death, or ICU transfer) were increasingly more frequent across the increasing 4C risk levels (Supplementary Table S2). However, when RDW was accounted for, considerable differences were observed regarding in-hospital mortality (predicted by this scoring system) within each risk level (Figure 3A): (i) at the “low risk” level (score 0–3), only 11 patients had RDW >normal—mortality was similar in normal- and high-RDW subsets (Figure 3A); (ii) however, at the “medium risk” level (score 4–8), mortality was 7.2% if RDW was normal (close to the expected 9.1–9.9% mortality), but it was 20.3% if RDW >normal (i.e., three-fold higher) (Figure 3A); (iii) at the “high risk” level (score 9–14), mortality was 25.1% if RDW was normal (somewhat less than expected 31.4–34.9%), but it was 39.0% if RDW was high (Figure 3A); (iv) mortality was higher if RDW high vs. RDW normal also in the “very high” risk subset (Figure 3A). All other mortality outcomes were higher if RDW was high than if normal at each level of 4C in-hospital mortality score (Figure 3A). Transfer to the ICU and the need for mechanical ventilation were also consistently numerically higher in RDW high vs. normal across the 4C score levels (Figure 3A). Similar patterns were observed in patients who started on remdesivir before deterioration or not (not shown). When investigating the predictive properties of 4C score, it provided overall good predictive properties of the investigated outcome (AUC 0.732, 95% CI (0.716–0.747)) when used as a sole parameter. Its properties were significantly improved when additionally accounting for elevated RDW (AUC 0.765, 95% CI (0.750–0.780)).

### 3.4. Accounting for RDW Improves VACO 30-Day Mortality Scoring System-Based Risk Stratification

Across the VACO levels of 30-day mortality risk (“low” [0–8.7%] to “extreme” [≥21.3%]), patients were significantly more likely to be progressively older, were less commonly started on remdesivir before deterioration, and had higher prevalence of high RDW and higher prevalence of higher 4C in-hospital mortality risk levels (Supplementary Table S3). Observed 30-day mortality was within the expectations at the “low” (6.6%) and “medium” VACO risk levels (14.2%), somewhat higher than expected at the “high risk” level (30.1%), and was 45.6% in the “extreme risk” patient subset (Supplementary Table S3). All other outcomes besides transfer to the ICU and mechanical ventilation were increasingly more common across the increasing VACO risk levels (Supplementary Table S3). However, when RDW was accounted for, considerable differences in 30-day mortality were obvious within a given VACO risk level: (i) in the “low”-risk subset, 30-day mortality was 5.4% (within expectations) if RDW was normal, but it was considerably higher (16.9%) if RDW was >normal (Figure 3B); (ii) in the “medium”-risk subset, mortality was 13.2% (within expectations), but it was considerably higher if RDW >normal (21.5%) (Figure 3B); (iii) in the “high”-risk subset, mortality was 26.3% (slightly higher than expected) (Figure 3B), but it was 45.8% if RDW >normal; (iv) in the “extreme”-risk subset, mortality was also much higher if RDW >normal than if normal (57.2% vs. 40.4%) (Figure 3B). All other outcomes were more common if RDW >normal than if normal within each stratum of the VACO risk level (Figure 3B). Similar patterns were observed in patients who started on remdesivir before deterioration or not (not shown). When investigating the predictive properties of the VACO index, it provided overall good predictive properties of the investigated outcome (AUC 0.725, 95% CI (0.709–0.740)) when used as a sole parameter. Its properties were significantly improved when additionally accounting for elevated RDW (AUC 0.754, 95% CI (0.740–0.769)).

## 4. Discussion

Since the beginning of the COVID-19 pandemic, a large number of studies have looked at the prognostic role of RDW in people affected by COVID-19 [20,21,22,23,24,25]. Numerous prognostic scores have also been used to assess the risk of clinical failure and mortality, but none have examined their significance in combination with RDW and whether it gives us any useful information for clinical practice, which is a novel contribution of our current study.

RDW is a non-specific hematological parameter with substantial inter- and intra-personal variability [26,27]. It is an indirect measure of the phenomenon of anisocytosis, i.e., having red blood cells of unequal size, and may help in quantifying the degree of anisocytosis. Variability in red blood cell size, hemoglobin content, and cholesterol content of erythrocyte membranes reflects the quality of hematopoiesis and can be affected by a large number of causes [28]. However, RDW seems to bear great prognostic potential that most likely reflects the pathophysiological mechanisms affecting hematopoiesis in the first place. These include nutritional deficiencies, most commonly iron deficiency, inflammatory processes, metabolic diseases, and primary hematologic diseases affecting the bone marrow, and diseases resulting in the damage and deformation of red blood cells through various mechanisms [29,30,31,32]. Different chronic diseases (considered comorbidities at the time of hospitalization for COVID-19 infection) utilizing various mechanisms may profoundly affect RDW at the time of hospital admission, on top of inflammatory stimuli introduced by COVID-19 itself. Despite criticism for using RDW (as well as other hematological indices) for obtaining prognostic information due to its non-specificity and variability, it should be noted that complete blood count (CBC)-derived indices consistently demonstrate superior prognostic properties, usually independently of established prognostic scores in cohorts of patients with various inflammatory diseases [33,34]. For example, models of machine learning that process large amounts of data have identified RDW as one of the parameters with the greatest impact on prognosis in patients with heart failure and polycythemia vera [35,36]. Considering especially COVID-19, several hematology-based risk scores have shown utility for improving prognostication, including leukocyte subsets and their ratios (neutrophil-to-lymphocyte ratio [37], monocyte count [38]), platelet count [39], anemia, polycythemia [40], RDW [23], etc. This is even more important considering that CBC is the first and foremost laboratory test for virtually all acutely ill patients and their treating physicians.

It should be noted that specific cut-offs for RDW elevation are probably context-specific, and different cut-offs bear maximal prognostic properties for specific purposes (for prognostication of various risks important for different diseases—thrombosis, bleeding, iron deficiency, mortality, respiratory deterioration, etc.). Also, various laboratories use different cut-off levels for normal ranges, mostly focusing on the healthy population, specific to the underlying population investigated. We decided to implement different cut-off levels for male and female patients, in line with other studies [19], as sex differences may play an important role in RDW variation. At the used cut-off points, RDW may be more useful for mortality prediction than for other outcomes (MV, ICU). It should also be noted that the implementation of sex-specific cut-off values allows for the identification of patients with RDW elevated due to more pronounced inflammatory phenomena and resulted in a higher proportion of male patients in the elevated RDW subgroup. This may contrast with the overall population and other COVID cohorts, where females are usually over-represented among patients with higher RDW.

Although a definitive mechanism for RDW elevation has not yet been established, our results are in agreement with other studies, and they argue that higher RDW is more likely in people who have chronic metabolic comorbidities, cardiovascular disease, chronic renal disease, chronic obstructive pulmonary disease, liver cirrhosis, active and metastatic malignancy, and dementia [13,22]. In our opinion, additional quantification of inflammation induced by comorbidities and COVID-19 itself, which is not encompassed by standard prognostic scores, is the most likely underlying cause of RDW-associated improvement in prognostication.

In discussing our results, it is important to mention that 4C and MEWSs are based on vital signs and clinical parameters associated with COVID-19, whereas the VACO index is based on parameters related to the patient regardless of the COVID-19 infection. RDW added to the 4C and VACO index can help in the better stratification of patients who are at higher risk of in-hospital and 30-day mortality, which can be explained by the fact that RDW is affected by comorbidities. The MEWS showed that proportions of those transferred to the ICU or requiring mechanical ventilation were also higher in patients with high vs. normal RDW, but the difference was small. In contrast to other scores, 4C seems to most accurately predict the outcome in the range of the expected frequencies. The MEWS and the VACO index, however, underestimated the frequency of outcomes compared to observed ones in the current cohort. However, RDW, as a low-cost parameter, allowed all the investigated prognostic scores to provide better information about patients who are at the highest risk of complications and thus may enable earlier identification and introduction of proper treatment to the most endangered patients. Among different scores, differences also exist in which risk subgroups RDW had the highest contribution. For example, the MEWS and VACO index RDW proved to be useful across the whole spectrum of risk categories, whereas no additional risk stratification was observed among 4C score low-risk patients. Exposure to remdesivir significantly differed across subgroups defined by RDW status and different prognostic scores. Notably, higher-risk patients assessed by clinical status (MEWS) were more frequently treated with remdesivir, whereas higher-risk patients assessed by comorbidities and RDW were less frequently treated with remdesivir. This is likely due to the presence of contraindications for remdesivir use associated with specific comorbidities. However, as our results suggest, RDW improved prognostication assessed by different scores regardless of remdesivir exposure.

The limitations of our study are single-center experience, retrospective study design, and the lack of a longitudinal assessment of RDW values. No causality of observed associations can be established due to the limitations of the study design. Given the dynamic nature of RDW in response to acute inflammation and treatment, longitudinal assessment could provide deeper insights into its role in patient outcomes, which could not be evaluated in the current study. The study population consists of patients of exclusively white race, thus limiting the global applicability of our findings. RDW is a non-specific marker affected by numerous conditions, and the current study could not control for nutritional status, specific hematologic conditions, etc. Some of the investigated prognostic scores were developed specifically for COVID-19 (VACO index, 4C score), whereas others were developed initially for other disease contexts (MEWS). The strengths of our study are the large sample size of patients with non-critical disease presentation, among whom timely and accurate prognostication regarding detrimental clinical course is of highest interest. Our results are representative of a large-volume referral center experience and may not be directly generalized to other clinical contexts. Considering the limitations, further validation of our findings is needed before wider adoption of RDW for the optimization of healthcare resource allocation.

## 5. Conclusions

RDW may be a useful tool for stratifying risk and prompting decisions, substantially improving prognostication when considered on top of commonly used prognostic scores in non-critical COVID-19 patients.

## Figures and Tables

**Table 1:** Key subject characteristics overall and stratified by red cell distribution width (RDW) level. Data are median (25th–75th percentile) or count (percent).

	All Patients	RDW Normal Range	RDW > Normal Range	*p* Value
N	3212	2584	628	-
Age	72 (63–82)	71 (62–81)	77 (68–84)	<0.001
Men	1804 (56.2)	1347 (52.1)	457 (72.8)	<0.001
X-ray pneumonia on admission	3101 (96.5)	2501 (96.8)	600 (95.5)	0.768
Started oxygen upon admission	2962 (92.2)	2377 (92.0)	585 (93.2)	0.308
Remdesivir before progression	566 (17.6)	494 (19.1)	72 (11.5)	<0.001
MEWS	2 (1–3)	2 (1–3)	2 (1–3)	0.109
MEWS 0–2	1765 (54.9)	1391 (53.8)	374 (59.5)	0.012
MEWS 3–4	1447 (45.1)	1193 (46.2)	254 (40.5)	0.012
4C score	11 (8–13)	10 (7–13)	13 (10–15)	<0.001
4C score 0–3 (low, 1.2–1.7%)	156 (4.9)	145 (5.6)	11 (1.7)	<0.001
4C score 4–8 (medium, 9.1–9.9%)	814 (25.3)	740 (28.6)	74 (11.8)	<0.001
4C score 9–14 (high, 31.4–34.9%)	1734 (54.0)	1393 (53.9)	341 (54.3)	0.819
4C score ≥15 (very high, 61.5–66.2%)	508(15.8)	306 (11.8)	202 (32.2)	<0.001
VACO index	16.4 (8.8–24.2)	14.7 (8.8–23.6)	21.3 (14.5–31.9)	<0.001
Low (0–8.7%)	707 (22.0)	630 (24.4)	77 (12.3)	<0.001
Medium (8.8–16.0%)	777 (24.2)	684 (26.5)	93 (14.8)	<0.001
High (16.1–21.2%)	671 (20.9)	540 (20.9)	131 (20.9)	0.976
Extreme (≥21.3%)	1057 (32.9)	730 (28.3)	328 (52.1)	<0.001
Need transfer to ICU	405 (12.6)	295 (11.4)	110 (17.5)	<0.001
Need mechanical ventilation	346 (10.8)	258 (10.0)	88 (14.0)	0.003
Died during hospitalization	879 (27.4)	591 (22.9)	288 (45.9)	<0.001
Died within 30 days	841 (26.2)	561 (21.7)	280 (44.6)	<0.001
Death or ICU within 60 days	939 (29.2)	633 (24.5)	306 (48.7)	<0.001
Charlson comorbidity index (CCI)	4 (2–6)	4 (2–5)	5.5 (4–7)	<0.001
CCI 0	181 (5.6)	170 (6.6)	11 (1.7)	<0.001
CCI 1–2	639 (19.9)	600 (23.2)	39 (6.2)	<0.001
CCI 3–4	1047 (32.6)	899 (34.8)	148 (23.6)	<0.001
CCI ≥5	1345 (41.9)	915 (35.4)	430 (68.5)	<0.001
Diabetes	1007 (31.4)	768 (29.7)	239 (38.1)	<0.001
Obesity	1004 (31.3)	819 (31.7)	185 (29.5)	0.273
Chronic heart failure	429 (13.4)	259 (10.0)	170 (27.1)	<0.001
Chronic renal failure	354 (11.0)	210 (8.1)	144 (22.9)	<0.001

RDW—red blood cell distribution width; MEWS—Modified Early Warning Score; ICU—intensive care unit; VACO—Veterans Health Administration COVID-19 Index; CCI—Charlson comorbidity index.

## Data Availability

Data are available from the corresponding authors upon reasonable request.

## Supplementary Materials

The following supporting information can be downloaded at: https://www.mdpi.com/article/10.3390/life14101267/s1, Figure S1: Probability of “death or intensive care unit (ICU) transfer within 60 days” predicted by the Modified Early Warning Score (MEWS) and probability of other outcomes by MEWS level (0-2 or 3-4) in respect to red cell distribution width (RDW), shown separately for patients treated and not-treated with remdesivir before deterioration; Table S1: Key subject characteristics stratified by the Modified early warning score (MEWS) level predicting the risk of a transfer to an intensive care unit (ICU) or death within 60 days; Table S2: Key subject characteristics across the levels of the 4C in-hospital mortality risk score and levels of the Veterans Health Administration COVID-19 (VACO) index 30-day mortality risk; Table S3: Key subject characteristics across the levels of the 4C in-hospital mortality risk score and levels of the Veterans Health Administration COVID-19 (VACO) index 30-day mortality risk.
