# Supplementary material for: Accounting for Red Cell Distribution Width Improves Risk Stratification by Commonly Used Mortality/Deterioration Risk Scores in Adult Patients Hospitalized Due to COVID-19
Source: Life (Basel). 2024 Oct 5;14(10):1267. doi: 10.3390/life14101267 (PMC11509295; doi:10.3390/life14101267)

Supplementary Table S1. Key subject characteristics stratified by the Modified early warning score (MEWS) level predicting the risk of a transfer to an intensive care unit (ICU) or death within 60 days. Data are median (25th – 75th percentile) or count (percent).

	MEWS 0-2	MEWS 3-4	P value
N	1765	1447	-
Age	75 (65-82)	70 (60-80)	<0.001
Men	950 (53.3)	864 (59.7)	<0.001
X-ray pneumonia on admission	1692 (95.9)	1409 (97.4)	<0.001
Started oxygen upon admission	1572 (89.1)	1390 (96.1)	<0.001
Remdesivir before progression	272 (15.4)	294 (20.3)	<0.001
RDW (%)	13.9 (13.3-14.9)	13.7 (13.2-14.6)	<0.001
RDW normal range	1391 (78.8)	1193 (82.5)	0.012
RDW ≥normal range	374 (21.2)	254 (17.5)	0.012
4C in-hospital mortality risk score	11 (8-13; 0-19)	10 (7-13)	0.007
4C score 0-3 (low, 1.2-1.7%)	70 (4.0)	86 (5.9)	0.009
4C score 4-8 (medium, 9.1-9.9%)	399 (22.6)	415 (28.7)	<0.001
4C score 9-14 (high, 31.4-34.9%)	1047 (59.3)	687 (47.5)	<0.001
4C score ≥15 (very high, 61.5-66.2%)	249 (14.1)	259 (17.9)	0.002
VACO index 30-day mortality risk	18.3 (10.9-26.7)	14.5 (6.5-22.6)	<0.001
Low (0-8.7%)	308 (17.5)	399 (27.6)	<0.001
Medium (8.8-16.0%)	401 (22.7)	376 (26.0)	0.723
High (16.1-21.2%)	402 (22.8)	269 (18.6)	0.006
Extreme (≥21.3%)	654 (37.1)	403 (27.9)	<0.001
Need transfer to ICU	184 (10.4)	221 (15.3)	<0.001
Need mechanical ventilation	156 (8.8)	190 (13.1)	<0.001
Died during hospitalization	444 (25.2)	435 (30.1)	0.002
Died within 30 days	426 (24.1)	415 (28.7)	0.003
Death or ICU within 60 days	470 (26.6)	469 (32.4)	0.017
Predicted probability of event[1]	7.9%	12.7%	<0.001
Charlson comorbidity index (CCI)	4 (3-6)	4 (2-5)	<0.001
CCI 0	65 (3.7)	116 (8.0)	<0.001
CCI 1-2	273 (15.5)	366 (25.3)	<0.001
CCI 3-4	605 (34.3)	442 (30.6)	0.024
CCI ≥5	822 (46.6)	523 (36.1)	<0.001
Diabetes	580 (32.9)	427 (29.5)	0.048
Obesity	531 (30.1)	473 (32.7)	0.100
Chronic heart failure	269 (15.2)	160 (11.1)	<0.001
Chronic renal failure	237 (13.4)	117 (8.1)	<0.001

RDW – red blood cell distribution width; MEWS – Modified Early Warning Score; ICU – intensive care unit; VACO – Veterans Health Administration COVID-19 Index; CCI – Charlson comorbidity index.

Supplementary Table S2. Key subject characteristics across the levels of the 4C in-hospital mortality risk score and levels of the Veterans Health Administration COVID-19 (VACO) index 30-day mortality risk. Data are median (quartiles) or count (percent).

	4C score 0-3	4C score 4-8	4C score 9-14	4C sore ≥15	P value
N	156	814	1734	508	-
Age	45 (37-48)	60 (55-66)	76 (69-82)	84 (80-88)	<0.001
Men	97 (62.2)	445 (54.7)	929 (53.6)	333 (65.6)	0.038
X-ray pneumonia on admission	143 (91.7)	774 (95.1)	1687 (97.3)	497 (97.8)	<0.001
Started oxygen upon admission	104 (66.7)	695 (85.4)	1661 (95.8)	502 (98.8)	<0.001
Remdesivir before progression	42 (26.9)	225 (27.6)	266 (15.3)	33 (6.5)	<0.001
RDW (%)	13.3 (12.9-13.9)	13.4 (12.9-14.0)	13.9 (13.3-14.9)	14.5 (13.8-15.6)	<0.001
RDW normal range	145 (92.9)	740 (90.9)	1393 (80.3)	306 (60.2)	<0.001
RDW ≥normal range	11 (7.1)	74 (9.1)	341 (19.7)	202 (39.8)	<0.001
MEWS score	3 (1-3)	3 (1-3)	2 (1-3)	3 (2-3)	0.423
MEWS score 0-2	70 (44.9)	399 (49.0)	1047 (60.4)	249 (49.0)	0.024
MEWS score 3-4	86 (55.1)	415 (51.0)	687 (39.6)	259 (51.0)	0.024
VACO 30-day mortality risk (%)	0 (0.0 -0.0)	6.5 (3.9-10.6)	18.8 (14.5-24.4)	29.5 (22.8-39.2)	<0.001
Low (0-8.7%)	156 (100)	466 (57.2)	84 (4.8)	1 (0.2)	<0.001
Medium (8.8-16.0%)	0	302 (37.1)	465 (26.8)	10 (2.0)	<0.001
High (16.1-21.2%)	0	28 (3.4)	557 (32.1)	86 (16.9)	<0.001
Extreme (≥21.3%)	0	18 (2.2)	628 (36.2)	411 (80.9)	<0.001
Need transfer to ICU	7 (4.5)	73 (9.0)	251 (14.5)	74 (14.6)	<0.001
Need mechanical ventilation	4 (2.6)	62 (7.6)	222 (12.8)	58 (11.4)	<0.001
Died during hospitalization	4 (2.6)	68 (8.4)	482 (27.8)	325 (64.0)	<0.001
Died within 30 days	4 (2.6)	68 (8.4)	482 (27.8)	325 (64.0)	<0.001
Death or ICU within 60 days	8 (5.1)	88 (10.1)	515 (29.7)	328 (64.6)	<0.001
Predicted probability of event[1]	1.2-1.7%	9.1-9.9%	31.4-34.9%	61.5-66.2%	<0.001
Charlson comorbidity index (CCI)	0 (0-0.7)	2 (1-3)	4 (3-6)	6 (5-8)	<0.001
CCI 0	117 (75.0)	61 (7.5)	2 (0.1)	1 (0.2)	<0.001
CCI 1-2	30 (19.2)	464 (57.0)	143 (8.2)	2 (0.4)	<0.001
CCI 3-4	8 (5.1)	226 (27.8)	752 (43.4)	61 (12.0)	0.321
CCI ≥5	1 (0.6)	63 (7.7)	837 (48.3)	444 (87.4)	<0.001
Diabetes	6 (3.8)	144 (17.7)	614 (35.4)	243 (47.8)	<0.001
Obesity	47 (30.1)	294 (36.1)	551 (31.8)	112 (22.1)	<0.001
Chronic heart failure	0	23 (2.8)	247 (14.2)	159 (31.3)	<0.001
Chronic renal failure	0	13 (1.6)	178 (10.3)	163 (32.1)	<0.001

RDW – red blood cell distribution width; MEWS – Modified Early Warning Score; ICU – intensive care unit; VACO – Veterans Health Administration COVID-19 Index; CCI – Charlson comorbidity index.

Supplementary Table S3. Key subject characteristics across the levels of the 4C in-hospital mortality risk score and levels of the Veterans Health Administration COVID-19 (VACO) index 30-day mortality risk. Data are median (quartiles) or count (percent).

	VACO low	VACO medium	VACO high	VACO extreme	P value
N	707	777	671	1057	-
Age	54 (48-58)	67 (64-70)	77 (72-81)	84 (79-88)	<0.001
Men	406 (57.4)	406 (52.2)	285 (42.5)	707 (66.9)	0.861
X-ray pneumonia on admission	680 (96.2)	753 (96.9)	647 (96.4)	1021 (96.6)	0.058
Started oxygen upon admission	622 (88.0)	718 (92.4)	629 (93.7)	993 (94.0)	<0.001
Remdesivir before progression	209 (29.6)	177 (22.8)	104 (15.5)	76 (7.2)	<0.001
RDW (%)	13.4 (13.0-14.3)	13.6 (13.0-14.3)	14.0 (13.4-15.2)	14.3 (13.6-15.3)	<0.001
RDW normal range	630 (89.1)	684 (88.0)	540 (80.5)	730 (69.1)	<0.001
RDW ≥normal range	77 (10.9)	93 (12.0)	131 (19.5)	327 (30.9)	<0.001
MEWS score	3 (2-3)	2 (1-3)	2 (1-3)	2 (1-3)	<0.001
MEWS score 0-2	308 (43.6)	401 (51.6)	402 (59.9)	654 (61.9)	<0.001
MEWS score 3-4	399 (56.4)	376 (48.4)	269 (40.1)	403 (38.1)	<0.001
4C score	5 (4-7)	9 (8-11)	12 (10-13)	14 (12-16)	<0.001
4C score 0-3 (low, 1.2-1.7%)	156 (22.1)	0	0	0	<0.001
4C score 4-8 (medium, 9.1-9.9%)	466 (65.9)	302 (38.9)	28 (4.2)	18 (1.7)	<0.001
4C score 9-14 (high, 31.4-34.9%)	84 (11.9)	465 (59.8)	557 (83.0)	628 (59.4)	<0.001
4C score ≥15 (very high, 61.5-66.2%)	1 (0.1)	10 (1.3)	86 (12.8)	411 (38.9)	<0.001
Need transfer to ICU	64 (9.1)	113 (14.5)	103 (15.4)	125 (11.8)	0.336
Need mechanical ventilation	49 (6.9)	96 (12.4)	97 (14.5)	104 (9.8)	0.253
Died during hospitalization	54 (7.6)	121 (15.6)	209 (31.2)	495 (46.8)	<0.001
Died within 30 days	47 (6.6)	110 (14.2)	202 (30.1)	482 (45.6)	<0.001
Death or ICU within 60 days	79 (11.2)	139 (17.9)	217 (32.3)	504 (47.7)	<0.001
Predicted probability of event[1]	0-8.7%	8.8-16.0%	16.1-21.2%	≥21.3%	<0.001
Charlson comorbidity index (CCI)	1 (0-2)	3 (2-4)	5 (4-6)	6 (5-7)	<0.001
CCI 0	179 (25.3)	1 (0.1)	0	0	<0.001
CCI 1-2	368 (52.1)	264 (34.0)	7 (1.0)	1 (0.1)	<0.001
CCI 3-4	129 (18.2)	404 (52.0)	311 (46.4)	203 (19.2)	<0.001
CCI ≥5	31 (4.4)	108 (13.9)	353 (52.6)	853 (80.7)	<0.001
Diabetes	137 (19.4)	222 (28.6)	257 (38.3)	391 (37.0)	<0.001
Obesity	279 (39.5)	293 (37.7)	201 (30.0)	231 (21.8)	<0.001
Chronic heart failure	17 (2.4)	36 (4.6)	106 (15.8)	270 (25.5)	<0.001
Chronic renal failure	26 (3.7)	40 (5.2)	77 (11.5)	211 (20.0)	<0.001

RDW – red blood cell distribution width; MEWS – Modified Early Warning Score; ICU – intensive care unit; VACO – Veterans Health Administration COVID-19 Index; CCI – Charlson comorbidity index.

Supplementary Figure S1: Probability of "death or intensive care unit (ICU) transfer within 60 days" predicted by the Modified Early Warning Score (MEWS) and probability of other outcomes by MEWS level (0-2 or 3-4) in respect to red cell distribution width (RDW), shown separately for patients treated and not-treated with remdesivir before deterioration. Probabilities (proportions) are given with Wilson 95% confidence intervals. MEWS risk levels are depicted by the MEWS score and expected (E(risk)) probability associated with the respective score. MV – mechanical ventilation.

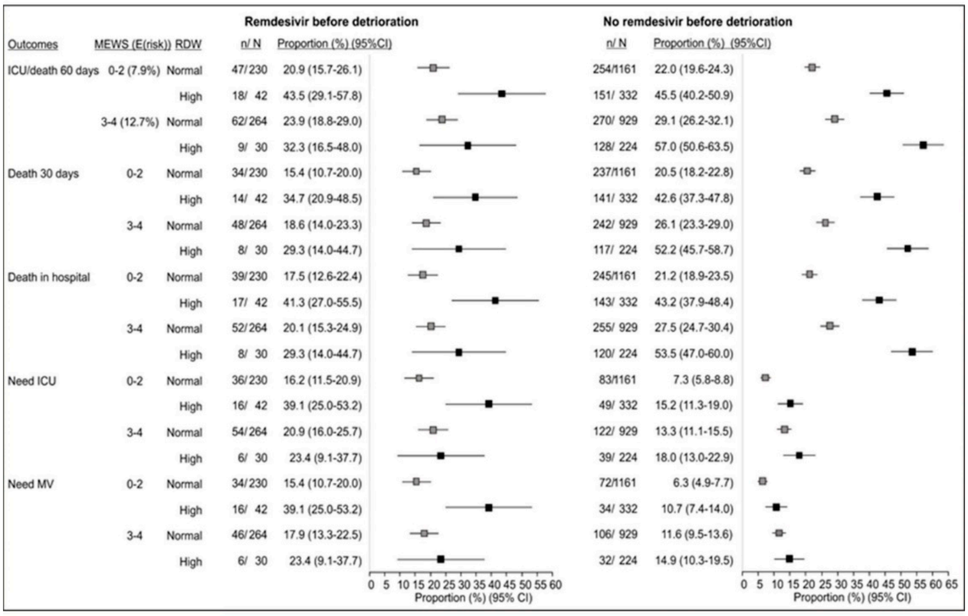

Supplement: Supplementary file 1 [file life-14-01267-s001.zip › life-3239037-supplementary.pdf]
